# Supplementary material for: CAR T-cell and oncolytic virus dynamics and determinants of combination therapy success for glioblastoma
Source: bioRxiv. 2025 Jan 25:2025.01.23.634499. Preprint. [Version 1] doi: 10.1101/2025.01.23.634499 (PMC11785192; doi:10.1101/2025.01.23.634499)
Supplement: Supplement 2 — S.2 Parameter value estimations Supplementary Table S.1: Parameter fitting for model (S.1). Average values of the parameters α,θTC,μ and δ from model (S.1), obtained from the fitting to the CAR T-cell and glioma cell data. The table is embedded in the Section S.2. Supplementary Table S.2: Parameter fitting for model (S.4). Average values of the parameter α and γ from model (S.4), obtained from the fitting to the OV and glioma cell data for burst size values b∈[0.025,2500] MOI/CI. The remaining parameter are fixed to K=5.2246 CI and ω=0.05h−1. Estimations of βTV are reported in Table S.4 and in Figure 5 in the main text. The table is embedded in the Section S.2. Supplementary Table S.3: Parameter fitting for model (1). Average values of the parameter α,βTV,θTC,θIC, and βCV from model (1), obtained by fitting to the CAR T-cells, OV, and glioma cell data. The remaining parameter are fixed to K=5.2246 CI, b=25 MOI/CI, ω=0.05h−1.γ is taken from the corresponding estimation in Table S.2, while δ and μ are taken from the corresponding estimations in Table S.1. The table is embedded in the Section S.2. [file media-2.pdf]

S.2 Parameter value estimations

We present the parameter values obtained from fitting the CAR T-cell module (S.1) (Table S.1), the OV module (S.4) (Table S.2), and the complete model described by system (1) in the main text (Table S.3) to the corresponding experimental data.

E:T	α (h^{-1})	θ_{TC} $(CI \cdot h)^{-1}$	μ $(CI \cdot h^{-1})$	δ (h^{-1})
1:10	0.2236	0.2085	0.026	0.014
1:25	0.2088	0.3965	0.0347	0.0761
1:50	0.1891	0.3640	0.0474	0.1094

Supplementary Table S.1: **Parameter fitting for model (S.1).** Average values of the parameters α, θ_{TC}, μ, and δ from model (S.1), obtained from the fitting to the CAR T-cell and glioma cell data.

V_0 [MOI]	α (h^{-1})	γ (h^{-1})
0.03	0.0571	0.0802
0.002	0.0508	0.0703
0.0008	0.0521	0.0335

Supplementary Table S.2: **Parameter fitting for model (S.4).** Average values of the parameter α and γ from model (S.4), obtained from the fitting to the OV and glioma cell data for burst size values $b \in [0.025, 2500]$ MOI/CI. The remaining parameter are fixed to $K = 5.2246$ CI and $\omega = 0.05$ h^{-1}. Estimations of β_{TV} are reported in Table S.4 and in Figure 5 in the main text.

E:T – V_0 [MOI]	α (h^{-1})	β_{TV} $(CI \cdot h)^{-1}$	θ_{TC} $(CI \cdot h)^{-1}$	θ_{IC} $(CI \cdot h)^{-1}$	β_{CV} $(CI \cdot h)^{-1}$
1:50 – 0.0008	0.1685	0.0886	0.3263	0.1741	0.0771
1:25 – 0.0008	0.2458	0.1099	0.4307	1.0992	0.0924
1:50 – 0.002	0.2444	0.0529	0.5897	1.3465	0.0672
1:25 – 0.002	0.3193	0.0917	0.6293	3.0165	0.0691

Supplementary Table S.3: **Parameter fitting for model (1).** Average values of the parameter α, β_{TV}, θ_{TC}, θ_{IC}, and β_{CV} from model (1), obtained by fitting to the CAR T-cells, OV, and glioma cell data. The remaining parameter are fixed to $K = 5.2246$ CI, $b = 25$ MOI/CI, $\omega = 0.05$ h^{-1}. γ is taken from the corresponding estimation in Table S.2, while δ and μ are taken from the corresponding estimations in Table S.1.
